# Effects of Exercise on the Oral Microbiota and Saliva of Patients with Non-Alcoholic Fatty Liver Disease

**DOI:** 10.3390/ijerph18073470

**Published:** 2021-03-26

**Authors:** Fumihiko Uchida, Sechang Oh, Takashi Shida, Hideo Suzuki, Kenji Yamagata, Yuji Mizokami, Hiroki Bukawa, Kiyoji Tanaka, Junichi Shoda

**Affiliations:** 1 Department of Oral and Maxillofacial Surgery, University of Tsukuba Hospital, Tsukuba, Ibaraki 305-8576, Japan; uchiyamada1031@yahoo.co.jp; 2 Division of Clinical Medicine, Faculty of Medicine, University of Tsukuba, Tsukuba, Ibaraki 305-8575, Japan; ohsechang@md.tsukuba.ac.jp; 3 Department of Medical Technology and Sciences, International University of Health and Welfare, Narita, Chiba 286-8686, Japan; t-shida@iuhw.ac.jp; 4 Department of Gastroenterology, Faculty of Medicine, University of Tsukuba, Tsukuba, Ibaraki 305-8575, Japan; hideoszk@md.tsukuba.ac.jp (H.S.); yuji-mizokami@md.tsukuba.ac.jp (Y.M.); 5 Department of Oral and Maxillofacial Surgery, Institute of Clinical Medicine, Faculty of Medicine, University of Tsukuba, Tsukuba, Ibaraki 305-8575, Japan; y-kenji@md.tsukuba.ac.jp (K.Y.); bukawah-cuh@umin.ac.jp (H.B.); 6 Faculty of Health and Sport Sciences, University of Tsukuba, Tsukuba, Ibaraki 305-8575, Japan; tanaka.kiyoji.ft@u.tsukuba.ac.jp

**Keywords:** periodontal disease, non-alcoholic fatty liver disease, exercise, clinical trial, oral microbiota, saliva components

## Abstract

Exercise can be hypothesized to play an important role in non-alcoholic fatty liver disease (NAFLD) treatment by changing the oral bacterial flora and in the mechanism underlying periodontal disease. We performed salivary component analysis before and after an exercise regimen, and genome analysis of the oral bacterial flora to elucidate the underlying mechanism. Obese middle-aged men with NAFLD and periodontal disease were allocated to 12-week exercise (*n* = 49) or dietary restriction (*n* = 21) groups. We collected saliva to compare the oral microflora; performed predictive analysis of metagenomic functions; and, measured the salivary immunoglobulin A, cytokine, bacterial lipopolysaccharide (LPS), and lactoferrin concentrations. The exercise group showed improvements in the clinical indices of oral environment. Salivary component analysis revealed significant reductions in LPS, and lactoferrin during the exercise regimen. Diversity analysis of oral bacterial flora revealed higher alpha- and beta-diversity after the exercise regimen. Analysis of the microbial composition revealed that the numbers of *Campylobacter* (+83.9%), *Corynebacterium* (+142.3%), *Actinomyces* (+75.9%), and *Lautropia* (+172.9%) were significantly higher, and that of *Prevotella* (−28.3%) was significantly lower. The findings suggest that an exercise regimen improves the oral environment of NAFLD patients by increasing the diversity of the oral microflora and reducing the number of periodontal bacteria that produce LPS and its capability.

## 1. Introduction

Periodontal disease has been shown to be associated with the onset and progression of metabolic syndrome [1,2,3]. Non-alcoholic fatty liver disease (NAFLD) can be regarded as a hepatic manifestation of the metabolic syndrome and it contributes to the risk of diabetic, neoplastic, cardiovascular, and, more generally, obesity-related morbidity and mortality. Notably, NAFLD is the most prevalent chronic liver disease in Japan [4]; moreover, diagnoses of concomitant NAFLD and periodontal disease are increasing rapidly worldwide [5].

Non-alcoholic steatohepatitis (NASH) is also referred to as “bacterial hepatitis”. Lipopolysaccharide (LPS), which is a component of the cell wall of Gram-negative bacteria, plays an important role in the multiple parallel hit hypothesis that was proposed by Tilg and Moschen [6], which has been proposed to be the mechanism underlying the development of NASH. Dysbiosis of the enteric bacteria, alongside systemic inflammatory and oxidative stress disorders that result from the greater absorption of LPS (i.e., metabolic endotoxemia), may contribute to the progression of chronic liver lesions.

In patients with NASH, LPS was previously considered to be derived solely from enteric bacteria; however, a relationship between NASH and periodontal bacterial infection has been reported in recent years [5,7,8]. *Porphyromonas gingivalis*, a highly toxic periodontal bacterium, is detected in 52% of saliva samples from patients with NASH [5]. According to medical epidemiologic data, which indicate that the prevalence of NASH and periodontal disease, have been dramatically increasing in recent years in Japan, LPS may not always be derived from enteric bacteria [9]. Therefore, periodontal bacteria may serve as a source of LPS and they play an important role in the pathogenesis of NASH.

To date, there has been no consensus regarding the prevention or treatment of NAFLD/NASH, except regarding the use of dietary and/or exercise regimens [10,11,12,13]. In addition, gingivitis and periodontitis are more severe in patients who undertake little physical activity. Thus, there appears to be a relationship between the level of physical activity and the onset of periodontal disease [14,15,16,17]. However, the studies that have shown that these associations were all cross-sectional in design and, therefore, provide poor evidence with which to guide treatment decisions. To the best of our knowledge, there have been no clinical studies to date that have analyzed the relationship between periodontal disease and exercise.

We have previously contributed to studies comparing the effects of dietary restriction and exercise regimens in obese middle-aged men with NAFLD [18,19,20,21]. In a series of clinical and animal studies [22], we showed that exercise: (a) ameliorated hepatic fat accumulation and inflammation/fibrosis [18]; (b) increased the phagocytic capacity of Kupffer cells [19]; (c) increased the hepatic clearance of LPS and reduced the innate immune response to LPS [22]; (d) activated the antioxidative stress-responsive transcription factor nuclear factor erythroid 2–related factor 2 (Nrf2) and increased the anti-inflammatory and oxidative stress responses [18]; and, (e) ameliorated periodontitis and reduced the populations of certain periodontal bacterial taxa [21]. These results suggest that oral bacteria are important in the development and progression of NAFLD, and that an exercise regimen may change the composition of the oral microflora and ameliorate periodontal disease.

In the present study, we aimed to analyze the residual saliva of previous study [21], conduct a metagenomic analysis of the oral microflora, and compare the metabolic activities of the microflora of obese patients with NAFLD and periodontal disease before and after a 12-week exercise regimen or a dietary regimen to define the changes in the oral environment and underlying any improvements.

## 2. Materials and Methods

### 2.1. Participants

**Figure 1:**
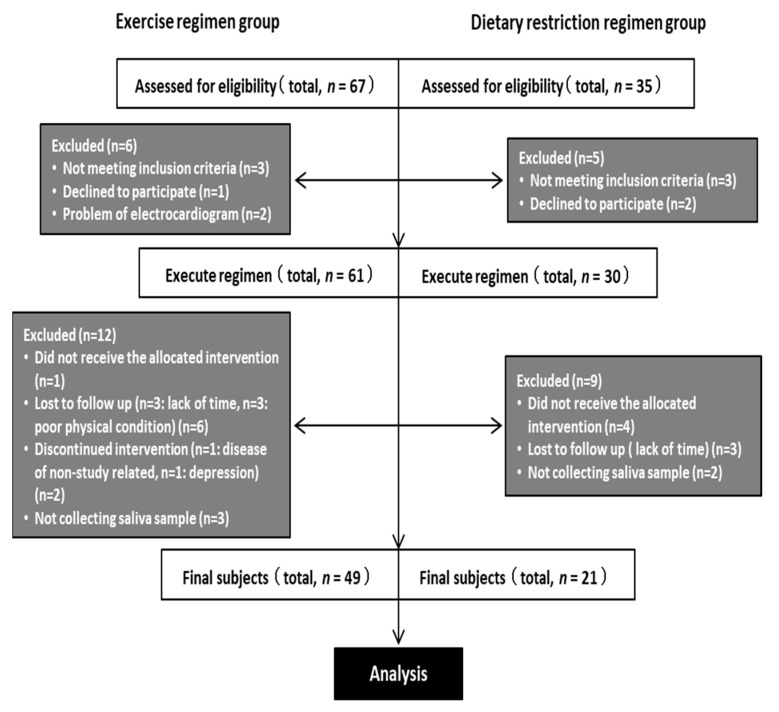
Flow chart for the study.

Figure 1 describes the workflow for the study. The participants in the study were recruited in Ibaraki Prefecture, Japan to participate in either an exercise or dietary restriction regimen, according to their preference, for 12 weeks between September 2014 and June 2015 at the University of Tsukuba. The participants were adult men who met the following criteria: body mass index (BMI) ≥ 25 kg·m^−2^ and <45 kg·m^−2^, concomitant NAFLD and periodontal disease [23], alcohol intake of <20 g·day^−1^, no daily exercise, no prior participation in any lifestyle modification regimen, a desire to establish appropriate exercise habits, an understanding of the methods and objectives of the study, and no ongoing medical treatment at the time of recruitment. The diagnosis of NAFLD was made based on the diagnostic criteria that were established by the diagnostic guidelines for NAFLD in the Asia–Pacific region [24].

Sixty-seven participants were recruited to the exercise regimen group (*E*) during the initial briefing session, of whom six were excluded: three did not meet the inclusion criteria, one refused to participate, and two exhibited abnormal electrocardiographic findings. Of these 61 participants, data from 49 were analyzed, following the further exclusion of 12 participants: one abandoned the program, three were lost to follow-up, three complained of sickness during the program period, two could not continue to participate in the regimen, and three could not participate in the post-exercise measurements or provide saliva for personal reasons (Figure 1).

Thirty-five participants were recruited to the dietary restriction regimen group (*D*) during the initial briefing session, of whom five were excluded: three did not meet the inclusion criteria and two refused to participate. Of the 30 remaining participants, the data from 21 were analyzed, following the exclusion of a further nine participants: four abandoned the program, three were lost to follow-up, and two could not participate in the post-intervention measurements or provide saliva (Figure 1).

Thus, of the original 102 participants, data from 70 (*E* group: *n* = 49 and *D* group: *n* = 21) were analyzed. Participants in both of the groups were instructed not to change their exercise or dietary habits other than as specified for each regimen during the study period. No dental guidance, treatment, and oral hygienic interventions was given or performed.

The study was approved by the Institutional Review Board of the University of Tsukuba (ID: H25-124, H25-156, and H26-118) and retrospectively registered in the University Hospital Medical Information Network Clinical Trial Registry (UMIN000022901). All of the procedures were performed in accordance with the principles of the Declaration of Helsinki. The objectives and design of the study were fully explained to all of the participants, who provided their written informed consent.

### 2.2. Exercise Regimen

The participants in the *E* group undertook an exercise program that consisted of 90 min. of exercise three times per week, under the guidance of a professional trainer. The exercise regimen comprised resistance exercise and aerobic exercise. The resistance exercise consisted of leg presses, leg extensions, leg curls, chest presses, seated rowing, pull-downs, and abdominal muscle exercises. The exercise volume was set at 170–190 kcal per session. The aerobic exercise mainly consisted of fast walking and/or light jogging for 20–40 min., with a maximal oxygen consumption (VO2max) of 60–85%, and the exercise volume was set at 180–360 kcal per session [21].

### 2.3. Dietary Restriction Regimen

Participants in the *D* group undertook a dietary restriction program that comprised a lecture or a consultation regarding their nutrition and dietary habits for 90 min. once per week with a registered dietitian. Food intake was recorded for each meal; the target intake was set at 560 kcal per meal and 1680 kcal·day^−1^ [20,25].

The program consisted of (a) instruction regarding nutritional balance, the modification of dietary behavior, and the weighing of food and energy intake calculation; (b) small interactive group sessions, during which participants could share their experiences, information, and ideas, to identify the most effective methods; and, (c) one-on-one counseling sessions to assist with personal goal setting and the management of physical problems and psychological distress, such that the participants could stick to their diet plans. The participants kept a food diary that the dietitians checked regularly, such that they could offer advice regarding appropriate food consumption and energy balance.

### 2.4. Saliva Sampling

The severity of periodontal disease was assessed before and after each regimen had been completed. Saliva production was stimulated by chewing paraffin gum for 3–5 min., and then the saliva was sampled using the discharge method. The samples were centrifuged at 1400× *g* at 4 °C for 10 min. and the supernatants were stored at −80 °C. Two hundred-microliter samples of saliva were transferred to microtubes and DNA extraction was performed using a DNeasy Blood & Tissue Kit (Qiagen, Venlo, Limburg, The Netherlands). The extracted DNA was stored at −30 °C until analysis. Oral examination and other measurements were performed one month before the intervention and one month after the intervention. There was a gap of 20 weeks between the 1st and 2nd sample collection. The measurements were conducted, as described previously [21].

### 2.5. Saliva Analysis

Commercial ELISA kits were used to determine the salivary concentrations of immunoglobulin A (IgA) (Salimetrics, Carlsbad, CA, USA) and lactoferrin (Abcam, Cambridge, MA, USA). The salivary lipopolysaccharide (LPS) concentration was determined using a *Limulus* Amoebocyte Lysate Assay Kit (Associates of Cape Cod, East Falmouth, MA, USA).

### 2.6. 16S rRNA Gene Sequencing

Two-step polymerase chain reactions were performed to obtain sequence libraries from purified DNA samples. The first polymerase chain reaction was performed using a 16S (V3–V4) Metagenomic Library Construction Kit for NGS (Takara Bio Inc., Kusatsu, Japan) with 341F (5′-TCGTCGGCAGCGTCAGATGTGTATAAGAGACAG-3′) and 806R (5′-GTCTCGTGGGCTCGGAGATGTGTATAAGAGACAG-3′) primers, which correspond to the V3–V4 region of the 16S rRNA gene. The second polymerase chain reaction was performed to add barcode sequences for Illumina sequencing while using a Nextera XT Index Kit (Illumina, San Diego, CA, USA). The prepared libraries were subjected to sequencing using a MiSeq Reagent Kit on a MiSeq (Illumina) at the Biomedical Center, Takara Bio.

### 2.7. Sequence Quality Control

The raw sequence data from the Illumina platform were converted into forward and reverse read files using the FASTQ processor (www.mrdnalab.com, accessed on 23 July 2019), which were then imported to Quantitative Insights Into Microbial Ecology (QIIME), an open-source microbiome analysis platform, for further analysis. The paired-end sequences were demultiplexed, and then denoised, dereplicated, and merged with the Cluster Database at High Identity with Tolerance Operational Taxonomic Unit (CD-HIT-OTU) quality control package in QIIME.

### 2.8. Microbiome Analysis

Sequence data processing, operational taxonomic unit (OTU) definition, and taxonomic assignment were performed using QIIME utilizing a naive Bayes classifier. The differential abundance of the OTUs at the different taxonomic levels, phylum, class, family, order, genus, and species, was analyzed using the analysis of composition of microbiomes (ANCOM). Alpha-diversity (observed species, Chao1, and Shannon phylogenetic diversity indices) was determined and analyzed using Wilcoxon’s rank sum test. The beta-diversity was estimated using the weighted UniFrac metric, which calculates the distances between the samples.

Changes in the biological functions that were associated with the microbiome were evaluated using Phylogenetic Investigation of Communities by Reconstruction of Unobserved States (PICRUSt) software [26] and the Kyoto Encyclopedia of Genes and Genomes (KEGG) database, release 70.0 [27]. Human-specific pathways were removed from the output to focus it on known bacterial pathways. The PICRUSt software uses 16S rRNA sequence profiles to estimate the metagenomic characteristics of the bacterial population, on the basis of the reference bacterial genomes and the KEGG pathway database. *p* < 0.05 was accepted as indicating statistically significant differences between groups.

### 2.9. Statistical Analysis

SPSS Statistics, version 23.0 (IBM Corp., Armonk, NY, USA) was used for statistical analysis. The data are presented as mean ± standard deviation. The Wilcoxon signed-rank test was used to analyze the longitudinal effects of each intervention and the Mann–Whitney *U*-test was used for comparing the *E* and *D* groups. *p* < 0.05 was considered to represent statistical significance.

## 3. Results

### 3.1. Anthropometric Characteristics

There were no significant differences in the baseline age, body mass, BMI, fat mass, or lean mass between the *E* and *D* groups (Table 1). Body mass, BMI, and lean mass significantly increased in the *E* group during the intervention, whereas fat mass significantly decreased (Table 2). In contrast, body mass, BMI, fat mass, and lean mass significantly decreased in the *D* group.

### 3.2. Biochemical Characteristics

The lactoferrin, LPS, and IgA concentrations significantly decreased in the *E* group, but not in the *D* group, during the intervention period, as shown in Table 2. The magnitude of the change in LPS concentration was greater in the *D* group than in the *E* group.

### 3.3. Alpha- and Beta-Diversity

**Figure 2:**
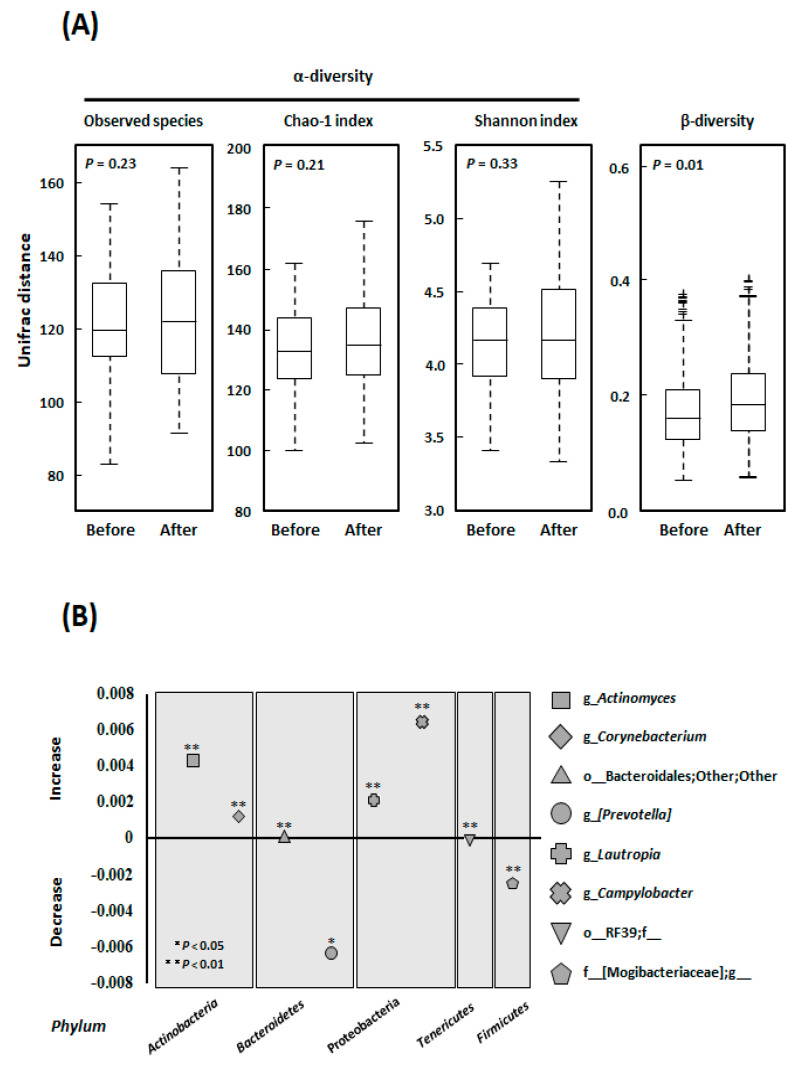
(**A**) Changes in the alpha-diversity and beta-diversity in the *E* group. The alpha-diversity indices (Observed species, Chao 1 index [operational taxonomic unit (out) richness estimation], and Shannon index [OTU evenness estimation]) and beta diversity indices were compared using Student’s unpaired t-tests (Bonferroni-corrected). (**B**) Comparative analyses of the taxonomic composition of the microbial community at the genus and higher levels. The populations of the genera *Actinomyces*, *Corynebacterium*, *Lautropia*, and *Campylobacter* significantly increased, as did that of the order *Bacteroidales* during exercise. In contrast, the populations of the genus *Prevotella*, order *RF39*, and family *Mogibacteriaceae* significantly decreased. * *p* < 0.05, ** *p* < 0.01 for baseline vs. three months in the *E* group (Student’s unpaired *t*-tests (Bonferroni-corrected)). “Other” denotes a taxonomic unit that has been isolated but not yet identified. No name after the character “_” denotes a taxonomic unit that has been identified but is still unnamed. A name in square brackets denotes a suggested name, which is based on analysis of the phylogenetic tree, but which is not yet verified, for an already identified taxonomic unit. Abbreviations: o, order; f, family; g_, genus.

We compared the alpha diversity of the oral microflora before and after the intervention in the *E* group while using Observed species, the Chao 1 index [OTU richness estimation], and the Shannon index [OTU evenness estimation]) (Figure 2A). Although all of the alpha-diversity metrics tended to increase during the interventions, there were no statistically significant differences (Observed species: *p* = 0.232, Chao 1 index: *p* = 0.210, and Shannon index: *p* = 0.328). In contrast, the beta-diversity significant increased (*p* = 0.010) (Figure 2A).

### 3.4. Microbial Community Composition at the Genus Level

The populations of the genera *Actinomyces* (+75.9%, *p* = 0.018), *Corynebacterium* (+142.3%, *p* = 0.001), *Lautropia* (+172.9%, *p* = 0.034), and *Campylobacter* (+83.9%, *p* < 0.001) were significantly increased by the exercise regimen, as was the size of the population of the order *Bacteroidales,* as shown in Figure 2B. In contrast, the populations of the genus *Prevotella* (−28.3%, *p* = 0.002), order *RF39* (−72.2%, *p* = 0.048), and family *Mogibacteriaceae* (−44.3%, *p* = 0.004) significantly decreased.

### 3.5. Differential Abundance of Bacterial Pathways

**Figure 3:**
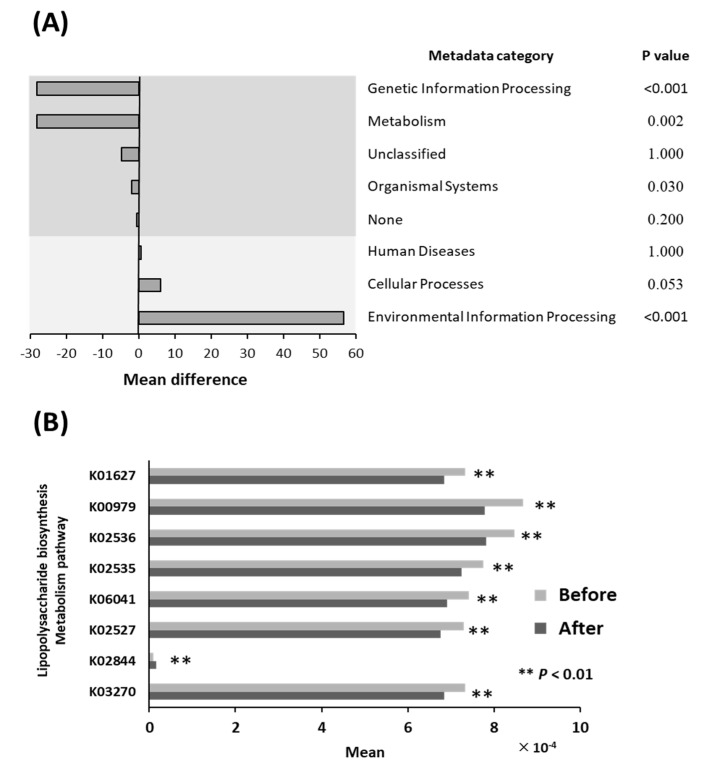
(**A**) Changes in the relative abundances of functional bacterial pathways in the *E* group. Potential differences (original value ×10^−4^) in microbiome functions were evaluated using PICRUSt software. The proportions of genes involved in Genetic Information Processing, Metabolism, and Organismal Systems significantly decreased during exercise. In contrast, the proportion of genes involved in Environmental Information Processing significantly increased. There were no significant differences in the proportions of genes that were Unclassified, with No known function, or those involved in Human Diseases or Cellular Processes. (**B**) Changes in the relative abundances of genes involved in lipopolysaccharide biosynthesis in the *E* group. For genome annotation in Kyoto Encyclopedia of Genes and Genomes (KEGG), the K number nodes were assigned to individual genes. In lipopolysaccharide biosynthesis pathways (https://www.genome.jp/kegg-bin/show_pathway?ko00540, accessed on 1 September 2020), the relative abundances of all the genes, except K02844, decreased during the exercise regimen.

Figure 3A shows the relative abundances of bacterial pathways before and after the intervention in the *E* group. The proportions of genes that were involved in Genetic Information Processing (−28.2 × 10^−4^, *p* < 0.001), Metabolism (−28.1 × 10^−4^, *p* = 0.002), and Organismal Systems (−2.0 × 10^−4^, *p* = 0.031) significantly decreased in the *E*. In contrast, the proportion of genes that were involved in Environmental Information Processing (+56.7 × 10^−4^, *p* < 0.001) significantly increased. There were no significant differences in the proportions of genes with Unclassified function (−4.8 × 10^−4^, *p* = 1), None function (−4.6 × 10^−5^, *p* = 0.200), or that are involved in Human Diseases (+7.2 × 10^−5^, *p* = 1) or Cellular Processes (+6.2 × 10^−4^, *p* = 0.053).

### 3.6. Lipopolysaccharide Biosynthesis Pathways

In the LPS biosynthesis and metabolism pathways, the relative abundances of all the genes were significantly reduced by the exercise regimen (K01627: −6.9%, *p* < 0.01; K00979: −10.2%, *p* < 0.01; K02536: −7.6%, *p* < 0.01; K02535: −6.5%, *p* < 0.01; K06041: −6.9%, *p* < 0.01; K02527: −7.3%, *p* < 0.01; and, K03270: −6.6%, *p* < 0.01), with the exception of K02844 (+83.0%, *p* < 0.01) (Figure 3B). The function of each gene is shown in the LPS biosynthesis pathway map (Figure S1).

## 4. Discussion

Following the observation that the exercise regimens restored hepatic pathological conditions of NAFLD, liver dysfunction, steatosis, and stiffness [19,21], in the present study we analyzed the saliva and performed metagenomic analysis of the oral microflora in obese middle-aged men with NAFLD and periodontal disease, before and after they participated in the exercise regimen. Metagenomic analysis of the oral microflora was not performed in a dietary regimen, because there was no change in clinical indices of periodontitis and populations of certain periodontal bacterial taxa [21]. The principal results were, as follows. (a) The exercise regimen reduced the salivary concentrations of LPS, lactoferrin, and IgA, whereas a dietary regimen did not. (b) Diversity analInserysis of the oral bacteria (alpha- and beta-diversities) revealed that the exercise regimen increased beta-diversity. (c) Analysis of the composition of the microflora revealed that the populations of *Campylobacter* (+83.9%), *Corynebacterium* (+142.3%), *Actinomyces* (+75.9%), and *Lautropia* (+172.9%) significantly increased, whereas that of *Prevotella* (−28.3%) significantly decreased, during the exercise regimen. (d) Metagenomic predictive analysis of bacterial functions revealed that the exercise regimen reduced the expression of genes that are involved in LPS biosynthesis in the oral bacteria. The expression of K02844 significantly increased during the exercise regimen; however, it was expressed at low levels and is involved in LPS biosynthesis in *Escherichia coli* (*E.coli*) and *Salmonella enterica* (*S. enterica*), as shown in Figure S1. These results suggest that an exercise regimen improves the oral environment in patients with NAFLD and periodontal disease, in contrast to the effects of a dietary regimen.

We previously performed an analysis of the changes in factors that are related to hepatic pathophysiology, including circulating organokine concentrations, phagocytosis by Kupffer cells, and the expression of target molecules of Nrf2 (an oxidative stress response transcription factor) in peripheral blood leukocytes in patients with obesity and NAFLD, which were caused by an exercise regimen [19]. Our results indicated that, although the accompanying reductions in body and fat mass may be minor, exercise appears to alter the production and secretion of various biologically active substances, and it has beneficial effects on the pathophysiology of.NAFLD. Exercise may also increase phagocytosis by Kupffer cells and induce anti-inflammatory/oxidative stress responses as a result of Nrf2 activation. Notably, exercise has also been shown to have specific physiologic effects, in addition to increasing energy expenditure, which are not caused by a dietary regimen [25].

The effect of exercise on Kupffer cells, which are liver macrophages, is noteworthy, because Kupffer cell function is impaired in patients with obesity, diabetes, or NAFLD/NASH [28]. The reduction in salivary LPS that is induced by the exercise regimen may be attributable to an increase in the capacity of oral macrophages to phagocytose LPS, as in Kupffer cells. Obesity is a chronic inflammatory disorder that is characterized by innate immune activity, which includes the activation of Kupffer cells and other immune mediators that eliminate unnecessary molecules. In patients with obesity, hyperleptinemia is associated with an excessive response to LPS, which involves the hyperactivation of inflammatory signaling pathways, including nuclear factor kappa-light-chain-enhancer of activated B cells [29]. Reductions in Kupffer cell function have been observed in both obese mice and a mouse model of NASH [30], and these are similar to the findings that were made in humans. When wild-type mice are subjected to moderate-to-intense exercise (continuous running) for approximately 12 weeks, they exhibit a greater clearance of exogenously-administered LPS [22] and an inhibition of LPS-induced inflammatory cytokine production. Furthermore, the elimination of the Kupffer cells does not increase LPS clearance or inhibit the production of inflammatory cytokines. These findings imply that exercise increases the capacity of Kupffer cells to phagocytose LPS and reduces the inflammation that is induced by LPS. The involvement of dehydroepiandrosterone, a steroid hormone that is induced by exercise, has been suggested to be part of the underlying mechanism [22].

**Figure 4:**
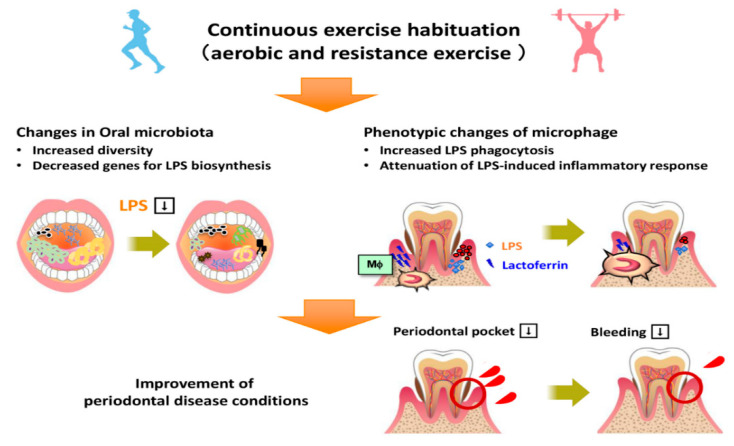
Summary of the mechanism by which the exercise regimen ameliorated periodontal disease. LPS, lipopolysaccharide.

Lactoferrin is an iron-binding glycoprotein that has a molecular weight of approximately 80,000 and a similar structure to that of transferrin [31]. Fecal lactoferrin content has been reported to be a useful biomarker of inflammatory bowel disease activity [32]. In addition, the concentration of lactoferrin in gingival sulcus fluid has been reported to be a marker of periodontitis [33]. In particular, patients with chronic periodontitis have higher concentrations of salivary lactoferrin than healthy individuals, and these concentrations are positively related to bleeding on probing and the number of sites with a probe depth of ≥6 mm. Thus, lactoferrin may represent a useful biomarker of inflammation in patients with periodontal disease. In the present study, exercise caused a reduction in the salivary lactoferrin concentration, alongside a reduction in the salivary LPS concentration. This suggests that exercise may inhibit the D group, since no change of indices were observed in production by increasing the diversity of the oral microflora, as described below, promote LPS clearance through an increase in macrophage activity [22], and reduce LPS-activated inflammatory signaling via the activation of an antioxidant stress response in vivo [18] (Figure 4).

The present findings imply that exercise increases the diversity of the oral microflora. This is noteworthy, because ulcerative colitis, an inflammatory bowel disease, has been reported to occur as a result of a loss of enteric bacterial diversity [34]; thus, periodontal disease may develop because of a loss of diversity of the oral microflora. The greater bacterial diversity may have contributed to an improvement in the oral environment by reducing the salivary LPS concentration and increasing the phagocytic capacity of the oral macrophages (Figure 4). Furthermore, it has been suggested that, in patients with periodontal disease, oral bacteria enter the gut and may affect the composition of the enteric bacterial community. Thus, it is increasingly clear that oral dysbiosis greatly affects the pathophysiology of hepatic disease, such as NAFLD, in patients with obesity, via a mouth-gut-liver axis [9].

This study had several limitations. First, with regard to the data that were collected for each intervention, it should be noted that this was not designed as a randomized controlled trial, and this is likely to lead to spurious causality and bias.

## 5. Conclusions

In conclusion, the following factors may have mediated the effects of exercise to ameliorate periodontal disease in patients with obesity and NAFLD: (a) an increase in oral bacterial diversity; (b) a reduction in the expression of genes that are involved in LPS biosynthesis by oral bacteria; (c) an increase in the phagocytic capacity of macrophages, and thus LPS clearance from the oral cavity; and, (d) reduction in the innate immune response to LPS and inflammation. In the future, the management of patients with obesity and NAFLD should involve attention to the oral environment. Moreover, exercise should be encouraged for its positive effects on the liver that occur via an amelioration of oral dysbiosis and a consequent improvement in the oral environment.

## Figures and Tables

**Table 1:** Anthropometric characteristics of the participants in each group at baseline.

Parameters	*E*	*D*	*E* vs. *D*
	*n* = 49	*n* = 21	*p*
Age	49.2 (8.3)	53.0 (9.9)	0.068
Body mass, kg	83.1 (13.5)	82.1 (10.5)	0.734
BMI, kg/m^2^	28.2 (3.9)	29.1 (2.4)	0.055
Fat mass, kg	21.4 (6.6)	20.9 (4.9)	0.989
Lean mass, kg	62.3 (8.4)	61.1 (7.9)	0.660

Values are presented as means  ±  standard deviation. *E* vs. *D* shows the comparisons of the baseline data between the exercise and dietary intervention groups. *E*, exercise regimen group; *D*, dietary restriction regimen group; and, BMI, body mass index.

**Table 2:** Anthropometric and biochemical characteristics of the participants at baseline and subsequent to the exercise and dietary regimens.

Parameters	*E* (*n* = 49)			*D* (*n* = 21)			*E* vs. *D*
	Before	After	*p*	Before	After	*p*	*p*
***Anthropometric characteristics***							
Age, year	49.2 ± 8.3			53 ± 9.9			0.068
Body mass, kg	83.1 (13.5)	83.6 (13.4)	0.026	82.1 (10.5)	72.8 (9.5)	<0.001	<0.001
BMI, kg/m^2^	28.2 (3.9)	28.4 (3.9)	0.024	29.1 (2.4)	25.9 (2.3)	<0.001	<0.001
Fat mass, kg	21.4 (6.6)	20.6 (6.6)	<0.001	20.9 (4.9)	15.9 (4.8)	<0.001	<0.001
Lean mass, kg	62.3 (8.4)	63.5 (8.1)	<0.001	61.1 (7.9)	56.6 (7.5)	<0.001	<0.001
***Biochemical characteristics***							
Lactoferrin, pg/mL	4743.7 (3200.2)	3826.3 (3375.7)	0.024	5350.7 (3226.4)	4133.1 (3466.8)	0.279	0.88
LPS, EU/mL	13,252.2 (11,941)	9959.5 (7417.1)	0.039	6919.8 (7799.5)	8423.5 (8524.4)	0.339	0.05
IgA, ug/mL	207.8 (182.9)	166.4 (142.2)	0.022	251.1 (99.4)	233.6 (142.0)	0.394	0.715

Values are presented as means (standard deviations). *E* vs. *D* shows the comparisons between the exercise and dietary intervention groups with respect to the changes from baseline to the 12-week end-point. BMI, body mass index; LPS, lipopolysaccharide; IgA, Immunoglobulin A.

## Data Availability

The datasets analysed during the current study are available from the corresponding author on reasonable request.

## Supplementary Materials

The following are available online at https://www.mdpi.com/article/10.3390/ijerph18073470/s1, Figure S1: Molecular functions represented in terms of functional orthologs: manually defined in the context of KEGG molecular networks, namely, KEGG pathway maps, BRITE hierarchies and KEGG modules.
